# Characterization of cognitive impairment in adult polyglucosan body disease

**DOI:** 10.1007/s00415-022-10960-z

**Published:** 2022-01-08

**Authors:** Paul Theo Zebhauser, Isabell Cordts, Holger Hengel, Bernhard Haslinger, Paul Lingor, Hasan Orhan Akman, Tobias B. Haack, Marcus Deschauer

**Affiliations:** 1 Department of Neurology, Technical University of Munich, School of Medicine, Munich, Germany; 2 Institute of Medical Genetics and Applied Genomics, University of Tübingen, Tübingen, Germany; 3 Department of Neurology, Columbia University Medical Center, New York, NY USA; 4 Centre for Rare Diseases, University of Tuebingen, Tuebingen, Germany

**Keywords:** Adult polyglucosan body disease, GBE1, Glycogen-branching enzyme, Cognitive impairment, Dementia

## Abstract

Adult polyglucosan body disease (APBD) is a rare but probably underdiagnosed autosomal recessive neurodegenerative disorder due to pathogenic variants in *GBE1*. The phenotype is characterized by neurogenic bladder dysfunction, spastic paraplegia, and axonal neuropathy. Additionally, cognitive symptoms and dementia have been reported in APBD but have not been studied systematically. Using exome sequencing, we identified two previously unreported bi-allelic missense *GBE1* variants in a patient with severe memory impairment along with the typical non-cognitive symptoms. We were able to confirm a reduction of GBE1 activity in blood lymphocytes. To characterize the neuropsychological profile of patients suffering from APBD, we conducted a systematic review of cognitive impairment in this rare disease. Analysis of 24 cases and case series (in total 58 patients) showed that executive deficits and memory impairment are the most common cognitive symptoms in APBD.

## Introduction

Adult polyglucosan body disease (APBD) is a rare autosomal recessive glycogenosis caused by bi-allelic variants in *GBE1*. Impaired glycogen-branching enzyme (GBE) activity and upregulation of glycogen synthase cause the accumulation of polyglucosan bodies with detrimental effects on neurons and glia in the central and peripheral nervous system. These pathophysiological events can result in a variety of neurological symptoms, which usually become clinically apparent around the age of 50 [1]. In a large case series, Mochel and colleagues [2] summarized the findings of 50 patients with APBD and found the most common symptoms to be neurogenic bladder dysfunction (100% of patients), spastic paraplegia, and axonal neuropathy with vibration loss (each 90%). Magnetic resonance imaging (MRI) brain image of these patients consistently revealed prominent white matter abnormalities in periventricular regions, the posterior limb of the internal and external capsule, the pyramidal tracts, as well as in the pons and the medulla.

Cognitive impairment over the course of the disease has been reported in a substantial proportion of patients but has not been systematically analyzed yet. In two larger observational studies of patients with APBD, mild cognitive impairment was found in about 50% of cases [2, 3]. However, a detailed investigation of affected cognitive domains or respective extent of impairments has not been performed. Over 25 years ago, Rifai and colleagues (1994) performed a non-systematic review of cognitive symptoms of 24 published APBD cases and observed varying cognitive deficits, mainly in the domain of memory, in about half of the reported cases. A substantial number of new cases of APBD have been published in the last decades, some of them explicitly addressing cognitive impairment in APBD and providing detailed but heterogeneous results of neuropsychological measurements [4–11].

Here, we present a patient with APBD with two novel bi-allelic missense variants in *GBE1* and prominent mnestic deficits along the typical non-cognitive symptoms of the disease. In addition, we provide a systematic review of reported cognitive impairment in studies and case reports of APBD in accordance with recent PRISMA guidelines [12] to more comprehensively compile the neuropsychological profile of patients suffering from this rare neurological disorder.

## Case report

A 68-year-old patient with a history of myocardial infarction, hypertension, and lumbar disc herniation was referred to our neurology clinic for further evaluation of progressive bladder dysfunction, gait disturbances, and cognitive deficits.

At the age of 60, the patient developed urge incontinence. About three years later, progressive gait instability with falls manifested. At presentation, the patient was mobile only with the help of walking sticks. As a very burdensome symptom with an insidious onset, he and his wife reported a slowly progressing cognitive decline over the past five to seven years, manifesting mainly in forgetfulness, problems with short-term memory, and a reduced ability to perform simple tasks simultaneously. Furthermore, his wife reported progressively cautious behavior in and toward unfamiliar surroundings and situations.

Neurological examination revealed marked symmetrical pallhypesthesia of the lower extremity, a positive Romberg’s test, and short-stepped unbalanced gait. Motor function and coordination, muscle tone, sensory function aside from pallesthesia, deep tendon reflexes (brisk besides weakened left ankle jerk reflex), cranial nerves, and bedside testing of higher cortical functions (aphasia, apraxia, visuospatial functioning) were normal.

Cerebrospinal fluid analyses showed only mild disturbance of the blood–brain barrier (elevated cerebrospinal fluid/serum albumin ratio of 12.3 × 10^3^). Analysis of amyloid and tau levels was normal for Amyloid beta 1–42, Amyloid beta 1–42/1–40 ratio, and p-181-tau. Total tau was slightly elevated (404 ng/l, cut off < 252), pointing to a neurodegenerative process without indication of Alzheimer’s disease.

**Fig. 1:**
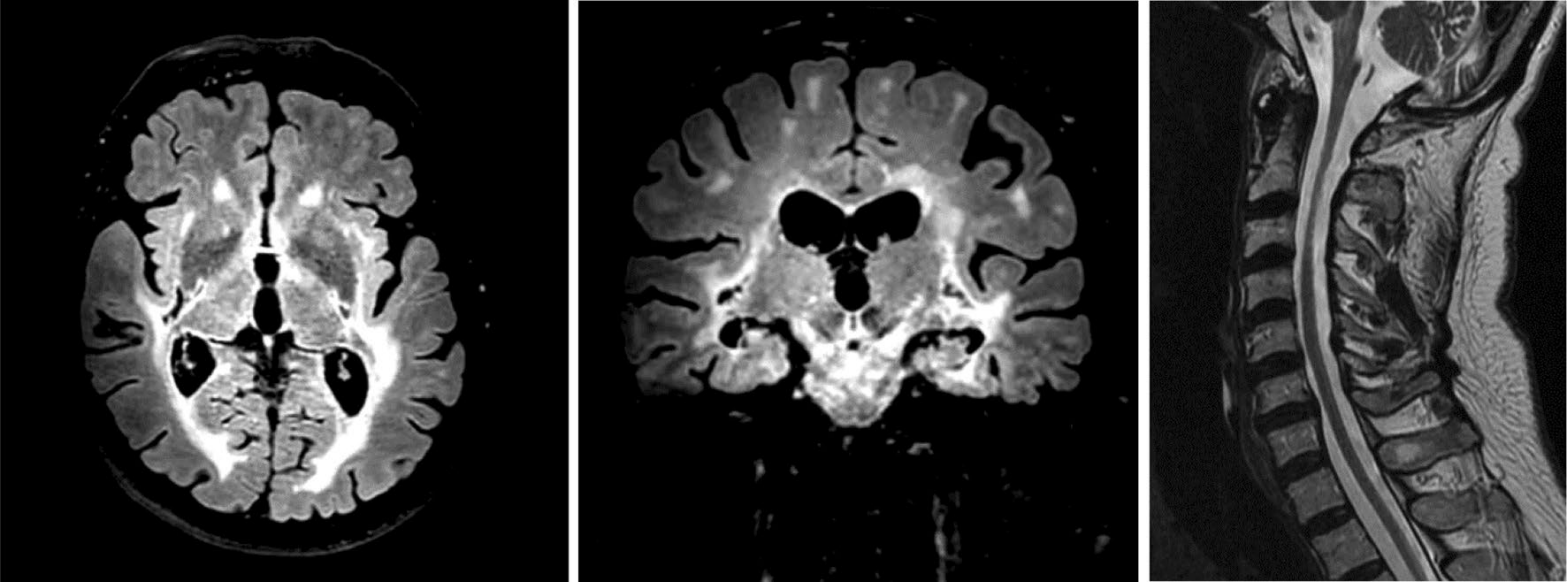
Cranial and spinal magnetic resonance imaging sections and MRI sequence from left to right—axial FLAIR, coronal FLAIR, and sagittal T2

Electroneurography and electromyography revealed a mild axonal polyneuropathy without myopathic signs. Cranial MRI revealed extensive bihemispheric leukencephalopathy with occipital predominance, including the pons and brainstem (Fig. 1). Furthermore, prominent global cerebral atrophy was present. Spinal MRI showed moderate cervical spinal cord atrophy.

### Neuropsychological testing

Formal neuropsychological testing showed severe deficits in verbal and visuospatial memory (encoding and recall). Executive functioning was not impaired and attentional functions were only slightly affected in a computerized test of phasic alertness (see Table 1 for a detailed depiction of performance on cognitive tests).

**Table 1:** Neuropsychological profile of case study

Cognitive domain (Test)	Percentile (sex-, age-, education-adjusted)
Attention	
Tonic alertness (Testbatterie zur Aufmerksamkeitsprüfung [13])	21
Phasic alertness (Testbatterie zur Aufmerksamkeitsprüfung)	10*
Processing Speed (Trail Making Test A; CERAD-Plus [14])	75
Executive functioning	
Cognitive Flexibility (Trail Making Test Ratio B/A)	50
Semantic fluency (“Animals”; CERAD-Plus)	16
Phonemic fluency (“S-Words”; CERAD-Plus)	62
Working memory verbal (digits backward; Wechsler Memory Scale [15])	57
Working memory visuospatial (block-tapping backward; Wechsler Memory Scale)	40
Memory	
Short-term memory (digits forward; Wechsler Memory Scale)	76
Short-term memory (visuospatial forward; Wechsler Memory Scale)	27
Verbal learning (word list; CERAD-Plus)	3*
Verbal learning—delayed recall (word list; CERAD-Plus)	< 1*
Visuospatial memory (recalling figures; CERAD-Plus)	< 1*
Visuospatial functioning	
Copying of figures (CERAD-Plus)	27
Other	
Boston Naming Test (CERAD-Plus)	79
Global Cognitive Functioning (MMSE [16])	< 1*

*CERAD* Consortium to Establish a Registry for Alzheimer’s Disease, *MMSE* Mini-Mental State Examination

*Numbers/percentiles in bold indicate below average performance

### Genetic analysis

Exome sequencing was performed as previously described [17]. Two novel heterozygous missense variants c.293 T > G (p.Val98Gly) and c.1753A > T (p.Arg585Trp) (GenBank: NM_000158.4) in *GBE1* were identified. Variant confirmation and carrier testing on available family members were conducted by Sanger sequencing. The patient’s three sons were each heterozygous for one of the two variants, indicating a compound heterozygous state of the variants in our patient. Both variants are very rare (c.293 T > G; minor allele frequency 4.43e-6) or absent (c.1753A > T) in gnomAD v2.1.1 (https://gnomad.broadinstitute.org/) and predicted to be deleterious in silico.

### Biochemical analysis: GBE activity

GBE activity was assayed in peripheral blood lymphocytes, as previously described [8]. Residual GBE activity was considerably decreased to 27.8% of normal (SD ± 27.2%; *n* = 3). These findings establish a functional relevance of the identified missense variants, which can be subsequently classified as likely pathogenic according to the recommendations of the American College of Medical Genetics and Genomics (ACMG) [18].

## Systematic review on cognitive impairment in APBD

**Fig. 2:**
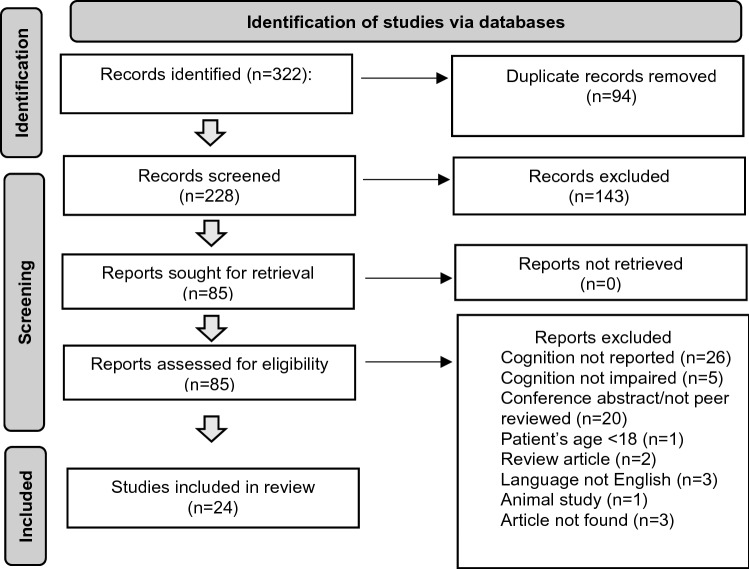
PRISMA flow diagram of study selection

We searched “Medline” via PubMed and “Web of Science Core Collection” via Web of Science (most recent search in October 2021, no historical limit applied) for published cases of APBD by applying the following search string: *(APBD OR (adult polyglucosan body disease))*. Review articles, conference abstracts, and articles in languages other than English were excluded. Furthermore, we excluded studies of patients under the age of 18. Aiming to characterize the profile and extent of cognitive impairments in APBD, we included all articles reporting on cognitive impairment in any modality (neuropsychological evaluation, cognitive screening, bedside testing, clinical impression). Additionally, we screened references of relevant articles. We identified a total of 58 patients from 24 case reports and series, which reported cognitive deficits in patients with APBD. Figure 2 depicts a PRISMA flow diagram of study selection. Of note, cognition was assessed but not impaired in 5 studies.

In Table 2, observational studies and cases with reports of cognitive impairment are listed and described.

**Table 2:** Overview of studies reporting cognitive impairments in APBD (in alphabetical order)

References	*n*	Sex, age	Clinical phenotype	Cognitive impairment	Note
[19]	1	m, 49	Gait disorder, hyperreflexia, pallhypesthesia, urinary incontinence	MMSE 16/30 with marked memory impairment	Severe clinical progress over 9 months
[20]	1	f, 32	Apathy, obsessive/compulsive behavior, inattention	MMSE 4/30, severe attention, and executive functioning deficits	Clinical FTLD, genetic analysis/neuropathological examination revealed APBD
[4]	1	f, 65	Memory impairment, apathy, urinary incontinence	MMSE 18/30, deficits in visual memory (learning and recall), apraxia, signs of dysexecutive behavior	Diagnosis of APBD based on cerebral and sural nerve biopsy (PBs)
[21]	2	m, 56	Spastic paraparesis, dysarthria, pallhypesthesia	No formal cognitive testing reported; “mild dementia” with forgetfulness	From a case series of 6 patients
		m, 87	Quadriplegic, fasciculations, bilateral Babinski	No formal cognitive testing reported; “possible mild dementia” with “memory difficulties”	
[5]	1	f, 54	FTLD	MMSE 20/30 (baseline) and gradually declining over 20 months to MMSE 0/30. Neuropsychological evaluation at baseline showed significant deficits in memory, executive functioning, language, visuospatial skills and apraxia	Neuropathological coexistence of APBD with FTLD
[22]	1	f, 57	Truncal ataxia, gait disturbances	No formal testing reported; “mild cognitive impairment” with word finding difficulties (progressive)	–
[23]	2	m, 57	Cerebellar syndrome, pallhypesthesia	No formal testing reported; “global intellectual impairment”	From a case series of 3 patients
		m, 74	Gait difficulties, urinary incontinence, pallhypesthesia	No formal testing reported; “global intellectual impairment” with memory loss and disorientation	–
[24]	1	m, 66	Ophthalmoplegia, bulbar palsy, sensory disturbance of legs, urinary incontinence	MMSE 19/30 with executive dysfunction	From a case series of 2 patients
[3]	14°/30	15m, 15f*	Spasticity in the legs (93%), hyporeflexia (100%), and bilateral pos. Babinski sign (100%), distal sensory deficit (80%)	No formal testing reported; “borderline to mild cognitive impairment in 47% of patients,” mostly frontal–subcortical impairment	23 of the patients were also included in Mochel et al. [2]*
[6]	1	m, 51	Urinary incontinence, spastic paraparesis, ataxic gait	Verbal fluency, mild deficits in visual attention, mild deficits in executive functioning	–
[25]	1	m, 70	Mild to moderate generalized weakness, sensorimotor PNP	MMSE 22/30 with impaired memory (delayed recall) and calculation	–
[7]	1	m, 38	Mild dysdiadochokinesia and bradykinesia, instable tandem gait	Moderate deficits in processing speed (severe), learning, memory (severe)	Cognition improved over 6 months of computerized working memory training
[26]	1	m, 48	Neurogenic bladder dysfunction	No formal testing reported; “mild cognitive dysfunction”	Imaging Study, no detailed clinical description provided
[8]	2	m, 64	Urinary incontinence, spastic paraparesis, symmetrical hyperreflexia, pallhypesthesia	Mild memory impairment, decreased performance on testing perseverance and response inhibition	–
		f, 58	Gait disturbance, urinary incontinence, mild spastic paraparesis with generalized hyperreflexia, pallhypesthesia	Mild memory impairment, naming difficulties with phonemic paraphasias, visual integration deficits, response disinhibition	
[27]	5	4m + 1f, 51–72	Spastic parapareses, urinary incontinence, hyporeflexia, distal sensory deficits (in 5/5 patients)	No formal testing reported; “mild cognitive impairment” in 3/5, “moderate cognitive impairment” in 2/5 patients	From a case series of 7 patients, of which two are reported elsewhere Lossos et al. [8]
[28]	1	f, 44	Dysarthria, dysphagia, atactic gait, dysmetric, dysdiadochokinesia	No detailed cognitive domains reported; “neuropsychological tests showed a severe cognitive impairment affecting both cortical and subcortical functions”	–
[2]	~ 25^/50	27m + 23m*§	Neurogenic bladder (100% of patients), spastic paraplegia with pallhypesthesia (90%), axonal neuropathy (90%)	No formal testing reported; mild cognitive decline with “attention deficit” in 24/50, “memory deficit” in 23/50 patients	–
[29]	1	m, 56	Spastic paraparesis, dysarthria, postural instability, pallhypesthesia	Severe deficits in visuospatial processing, apraxia, verbal abilities, sustained attention, memory/verbal learning, and visuospatial abilities (copying and visual organization)	–
[9]	1	f, 50	Parkinsonism, pallhypesthesia, hyporeflexia	Immediate and delayed recall, verbal fluency, executive functioning, arithmetic abilities	Diagnosis of APBD based on sural nerve biopsy
[30]	2	m, 59	Wide-based gait and spastic lower limbs, neurogenic bladder	No formal testing reported; “poor recent memory”	From a case series of 4 patients
		f, 64	Spastic paraplegia, bilateral Babinski signs, pallhypesthesia	No formal testing reported; “poor memory”	
[31]	1	m, 44	Ataxic-spastic gait, nystagmus, dysarthria, pallhypesthesia	No formal testing reported; “memory deficits and difficulty in planning”	–
[10]	1	f, 61	Dysdiadochokinesis, gait disturbances,	Widespread moderate to severe cognitive impairment in memory, language, executive functioning, and visuospatial abilities	Repeated neuropsychological testing over 33 months: no progression
[32]	1	m, 69	Hyperreflexia, positive Babinski sign bilaterally	MMSE 24/30, deficits in visuospatial abilities, working memory, apraxia, severe deficits in delayed verbal recall, word fluency (semantic and phonematic)	–
[11]	1	m, 62	Gait instability, bladder dysfunction, proximal weakness, distal sensory loss	No formal testing reported; progressive cognitive deficits with “poor judgment, inability to concentrate, and short-term memory loss”	–

*n* number, *f* female, *m*  male, *MMSE* Mini-Mental State Examination [16], *FTLD* frontotemporal lobar degeneration, *PBs* polyglucosan bodies, *PNP* polyneuropathy

*Patients age provided only with regard to selected symptoms, ° = in 14/30 patients cognitive deficits were reported; as 23/30 patients from this study were reported elsewhere (Mochel et al. [2]) we estimate the number of newly reported patients with cognitive symptoms to be *n* = 2, ^ = authors state that “up to half of the patients” were affected by cognitive symptoms; based on that we estimated the number of patients with cognitive symptoms to be *n* = 25, § = exact number of patients with cognitive deficits cannot be determined based on data provided

Extensive neuropsychological evaluation was performed in 11 patients. Memory impairment and executive dysfunction were identified in 9 patients, each with a large overlap of these symptoms (memory and executive deficits observed in 7 patients). Attentional deficits were observed in 4 patients only. When including studies with less advanced evaluation of cognitive impairment, this relation remained constant. Detailed results of respective neuropsychological studies can be found in Table 2.

**Fig. 3:**
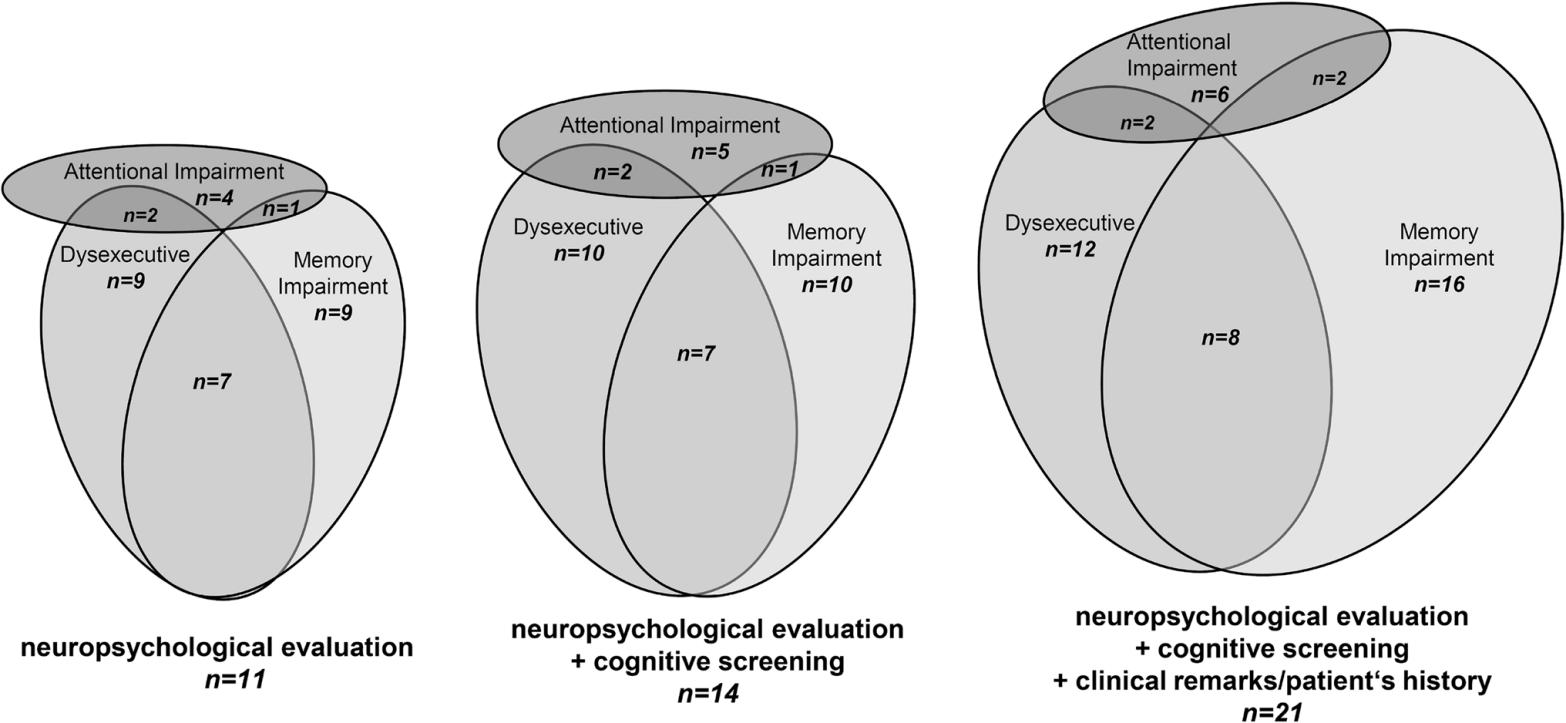
Proportional Venn diagrams of reported cognitive impairment in studies of patients with APBD split by method of evaluation. Only studies from Table 2 which reported cognitive impairments in specific domains (attention, executive functioning, memory) are included

Figure 3 summarizes these findings focusing on three major cognitive domains (attention, executive functioning, memory) using proportional Venn diagrams to illustrate the overlap of affected domains, split by quality of the reported cognitive assessment (detailed neuropsychological evaluation, brief cognitive screening, or clinical remarks/patient’s history). Furthermore, a variety of cortical symptoms were noted in a few patients (apraxia *n* = 3, language difficulties or aphasia *n* = 2, dyscalculia *n* = 2, and visuospatial impairments *n* = 2).

## Discussion

In this study, we present a case of APBD with severe multimodal and isolated memory deficits along with the typical clinical symptoms and MRI findings of the disease. Genetically, we identified two previously unreported bi-allelic missense *GBE1* variants and confirmed functional relevance by detection of a decreased GBE activity in blood lymphocytes.

While the triad neurogenic bladder dysfunction, spastic paraplegia (which was absent in our patient although brisk reflexes were observed), and axonal neuropathy can be observed in the vast majority of patients with APBD [2, 3], cognitive symptoms have not been sufficiently studied yet. However, in clinical practice, cognitive impairments in neurological disorders regularly pose a substantial problem for patients and caregivers, often going beyond the effects of motor or sensory deficits [33, 34].

Here, we provide a systematic evaluation of cognitive impairment in APBD by conducting a systematic review of 24 case reports and case series of APBD, including 58 patients with reported cognitive symptoms. In patients who underwent neuropsychological testing, memory and executive functioning were the most commonly affected domains (81% of patients, respectively). This pattern persisted after the inclusion of studies that only performed brief cognitive screening or reported clinical remarks or patients’ histories regarding cognitive symptoms.

Based on the reported studies and their respective nature (mainly case reports), it is difficult to determine the prevalence of cognitive impairment in APBD. In our study, the rationale was not to characterize the frequency of cognitive impairment in this condition. As opposed to the 24 studies which found cognitive deficits in patients with APBD, we found only five studies reporting normal cognitive functioning. This might reflect diagnostic bias, as in clinical practice, extensive neuropsychological testing is often only carried out in overt cases of cognitive impairment. Based on two larger case series [2, 3], it was estimated that approximately 50% of patients showed cognitive symptoms. However, to our knowledge, no systematic screening of cognitive functioning or formal neuropsychological evaluation has been conducted in a series or larger sample of patients with APBD. To determine the frequency and precise profile of cognitive deficits in APBD, larger studies or case series are needed. Also, longitudinal studies on cognitive impairment in APBD would be of interest to better understand the course of cognitive symptoms in this disease. Presumably, cognitive deficits occur later in the course of the disease, as it is the case in a variety of neurodegenerative movement disorders (e.g., Parkinson’s disease). For clinical practice, we strongly recommend neuropsychological testing in patients suffering from APBD to identify even subtle or subclinical deficits. Being informed about attentional, executive or mnestic deficits are of significant value for caregivers and patients, as even mild deficits can have profound effects on abilities of daily living, social life, and working ability. Furthermore, detailed neuropsychological evaluation enables patient-tailored cognitive training, which has been shown to be effective in APBD in one patient [7].
